# Refining the Content of the AGEC Accreditation Handbook

**DOI:** 10.1093/geroni/igab046.161

**Published:** 2021-12-17

**Authors:** Donna Schafer, Donna Weinreich

**Affiliations:** 1 National Association for Professional Gerontologists, Healdsburg, California, United States; 2 Western Michigan University, Plainwell, Michigan, United States

## Abstract

The AGEC Accreditation Handbook and Self-Evaluation Guide has been revised several times since the first drafts were produced in 2016 and 2017. The Handbook contains information for accreditation applicants, AGEC reviewers, and members of the Board of Governors responsible for administering AGEC policies and procedures. It includes the accreditation standards, eligibility for accreditation, timelines, and self-study and site visit guidelines. It is the central document for the AGEC accreditation process. Handbook revisions, drawn from the experience of accrediting programs, have been for the purposes of clarifying expectations and simplifying the application process. This presentation highlights modifications included in the 2020 Handbook, which simplify the self-study outline and site visit guidelines, and the 2021 modifications, which clarify the “Mapping Course Matrix” (displaying AGHE competencies covered in program courses) and add an “Assessment Matrix” for measuring acquisition of the competencies and improving the program as needed.

